# CoverView: a sequence quality evaluation tool for next generation sequencing data

**DOI:** 10.12688/wellcomeopenres.14306.1

**Published:** 2018-04-04

**Authors:** Márton Münz, Shazia Mahamdallie, Shawn Yost, Andrew Rimmer, Emma Poyastro-Pearson, Ann Strydom, Sheila Seal, Elise Ruark, Nazneen Rahman

**Affiliations:** 1 Division of Genetics & Epidemiology, The Institute of Cancer Research, London, SM2 5NG, UK; 2 TGLclinical, The Institute of Cancer Research, London, SM2 5NG, UK; 3 Cancer Genetics Unit, Royal Marsden NHS Foundation Trust, London, SM2 5PT, UK

**Keywords:** NGS, Quality Sequencing Minimimum, QSM, quality assurance, quality control, depth of coverage, base quality, mapping quality, genetic testing

## Abstract

Quality assurance and quality control are essential for robust next generation sequencing (NGS). Here we present CoverView, a fast, flexible, user-friendly quality evaluation tool for NGS data. CoverView processes mapped sequencing reads and user-specified regions to report depth of coverage, base and mapping quality metrics with increasing levels of detail from a chromosome-level summary to per-base profiles. CoverView can flag regions that do not fulfil user-specified quality requirements, allowing suboptimal data to be systematically and automatically presented for review. It also provides an interactive graphical user interface (GUI) that can be opened in a web browser and allows intuitive exploration of results.

We have integrated CoverView into our accredited clinical cancer predisposition gene testing laboratory that uses the TruSight Cancer Panel (TSCP). CoverView has been invaluable for optimisation and quality control of our testing pipeline, providing transparent, consistent quality metric information and automatic flagging of regions that fall below quality thresholds. We demonstrate this utility with TSCP data from the Genome in a Bottle reference sample, which CoverView analysed in 13 seconds.

CoverView uses data routinely generated by NGS pipelines, reads standard input formats, and rapidly creates easy-to-parse output text (.txt) files that are customised by a simple configuration file. CoverView can therefore be easily integrated into any NGS pipeline. CoverView and detailed documentation for its use are freely available at github.com/RahmanTeamDevelopment/CoverView/releases and www.icr.ac.uk/CoverView

## Introduction

Next generation sequencing (NGS) has transformed genetic medicine by dramatically reducing the cost and time of genetic testing, which has led to a rapid global expansion in clinical genetic testing ^1^. It is obviously crucial that these tests are accurate, with low false positive and false negative error rates. Robust quality assurance and quality control are required to minimise these errors and thus ensure reliable test results ^2^.

Three primary metrics are used to evaluate sequence quality in NGS data: depth of coverage (how many sequence reads are present at a given position), base quality (have the correct bases been called in sequence reads) and mapping quality (have the reads been mapped to the correct position in the genome) ^3^. False negative errors are often caused by insufficient depth of coverage ^4^, and it is vital that regions with low coverage are flagged and reviewed, not least because they may require additional interrogation ^5^. Coverage evaluation is also useful for comparing different NGS library generation strategies, to identify regions with suboptimal performance ^6, 7^ and for probe design optimisation ^8^.

Poor base and poor mapping quality can cause false negative and false positive errors. Systematic base calling errors often occur in regions with high or low GC content or in homopolymer stretches ^9^, while mapping errors often occur in regions of high homology (e.g. pseudogenes) that result in ambiguously mapped reads ^10^. As a starting point for evaluating base and mapping quality, base callers and read mappers provide Phred quality scores that quantify base and mapping quality. These scores give the probability that a particular base has been identified incorrectly (base quality score, BQ ^11^) or a read has aligned to the wrong genomic position (mapping quality score, MQ) ^12^.

Several tools and packages for analysing depth of coverage, base and mapping quality exist, for example GATK DepthOfCoverage and DiagnoseTargets ^13^, QualiMap ^14^, FastQC ^15^, samtools depth ^16^ and pysamstats ^17^. These tools have different strengths, weaknesses and trade-offs, particularly in relation to flexibility and usability. Most available tools were developed for the research environment rather than the medical setting and have not focused on the needs and requirements of genetic testing in patients.

Here we present CoverView, a quality evaluation tool for NGS data that was designed to be user-friendly, fast, flexible and easy to integrate into NGS analysis pipelines. CoverView was developed to provide the quality assurance and quality control information required by clinical NGS testing laboratories, though we believe it is equally useful for research use. We recently proposed the Quality Sequencing Minimum (QSM) to deliver comprehensive, consistent, transparent NGS quality assurance information about depth of coverage, base and mapping quality, and we use CoverView to evaluate fulfilment of a QSM in our laboratory ^8^. We also use CoverView as the quality control tool for all our research and clinical NGS analyses and it is integrated into our exome analysis tool, OpEx (Optimised Exome) ^18^.

## Methods

### Implementation

CoverView is implemented as an easy-to-use tool that can process the read count, BQ and MQ of mapped sequencing reads. It reports a series of informative quality control (QC) metrics with increasing levels of detail from a chromosome-level summary to per-base profiles. It also flags regions that do not pass user-defined quality requirements. The tool is implemented in Python v.2.7.13 and Cython v.0.25.2, with its graphical user interface (GUI) developed in Flask v. 0.12.1, HTML5 v.5.1 and JQuery v.3.1.1.

CoverView requires a BAM file (containing the mapped reads) as its input with the corresponding .BAI file ^16^. A BED file is also required with each record in the BED file defining the user-specified genomic region of interest (e.g. an exon of a gene) for which depth of coverage and sequence quality metrics will be reported. The BAM file may optionally contain reads marked as duplicates as CoverView can generate metrics with duplicate reads either included or excluded.

CoverView generates four output (.txt) files that provide different information about the quality of the input BAM dataset (for CoverView input files see *Data and software availability* section) ^19^. At the most detailed level, per-base profiles of position specific metrics are reported for each region ( Table 1). This base-level resolution is important because regions may only partially fail quality metrics, for example one part of an exon may have high quality depth of coverage whilst another part is poorly covered.

**Table 1.** Position specific metrics reported as per-base profiles in the specified genomic regions.

Position specific metrics	Definition
Coverage (COV)	The number of mapped reads covering the position
Median Base Quality (MEDBQ)	Median base quality of all read bases mapping to the position
Fraction of Low Base Quality (FLBQ)	Fraction of read bases mapping to the position with a base quality lower than a user-specified threshold
Median Mapping Quality (MEDMQ)	Median mapping quality of all reads covering the position
Fraction of Low Mapping Quality (FLMQ)	Fraction of reads covering the position with a mapping quality lower than a user-specified threshold
Quality Coverage (QCOV)	Number of mapped reads covering the position with read mapping quality and base quality higher than user-specified threshold

The coverage profile (COV) provides information on how the depth of coverage changes across the region, whilst the FLBQ and FLMQ metrics describe the fraction of coverage of low base or mapping quality scores at each position, respectively ( Table 1). This is important because at positions with high FLBQ or FLMQ values it may not be possible to call variants with confidence even if the COV threshold is met. In addition, mapping and base quality scores are summarised by their median in the per-base MEDBQ and MEDMQ profiles. Finally, the QCOV profile integrates coverage, base and mapping quality information for each base by counting the number of covering reads that satisfy the user-defined quality requirements.

As systematic base calling errors can be strand-specific ^20^, QC metrics for forward and reverse reads separately can be of interest. CoverView can, optionally, output the described profiles calculated for forward (+) and reverse (-) reads only, facilitating detection of strand-specific biases in coverage or base and mapping quality.

Summary statistics derived from the per-base profiles are reported for each user-specified region to describe the overall quality of the region ( Table 2) ^19^. MEDCOV and MEDQCOV summarise the coverage profiles by their median across positions and MINCOV and MINQCOV provide information about the least covered position. Finally, the MAXFLMQ and MAXFBLQ metrics describe the lowest sequence quality positions in the region. CoverView can also output region-level metrics calculated for forward and reverse reads separately.

**Table 2.** Summary metrics for the specified genomic regions.

Summary metrics of region	Definition
Read count (RC)	Total number of mapped reads overlapping the region
Median coverage (MEDCOV)	Median of COV values across all positions in the region
Minimum coverage (MINCOV)	Minimum of COV values across all positions in the region
Median quality coverage (MEDQCOV)	Median of QCOV values across all positions in the region
Minimum quality coverage (MINQCOV)	Minimum of QCOV values across all positions in the region
Maximum fraction of low mapping quality (MAXFLMQ)	Maximum of FLMQ values across all positions in the region
Maximum fraction of low base quality (MAXFLBQ)	Maximum of FLBQ values across all positions in the region

Users can define the minimal requirements to ‘pass’ the quality test and if this is not met the region of interest is ‘flagged’. Defining these minimum requirements for depth of coverage, base and mapping quality are the basis of the QSM that is described in detail in the accompanying paper ^8^. In CoverView a minimum or maximum value can be specified for any of the metrics in Table 2. For example, users may set a lower threshold to MINCOV and an upper threshold to MAXFLBQ: i.e. a region will be tagged with “FLAG” if coverage is below the defined value or FLBQ exceeds the maximum value at any position within the region, otherwise the region will be tagged as “PASS”. A separate column in the output file indicates the pass/flag status of each region.

For correct variant annotation, interpretation and follow-up it is essential to know which gene transcripts a flagged region overlap with. A simple script (ensembl_db) is included for creating a transcript database using any Ensembl release. In a separate output file, CoverView optionally reports the transcript coordinates (CSN coordinates ^21^) of all genomic intervals that are covered by less than 15 high quality reads (QCOV<15), which are referred to as “poor quality intervals” ^19^. Users may wish to review positive or negative calls within such regions, to ensure confidence in the calls.

CoverView also provides a chromosome-level summary ^19^. This is important because the input BAM file may contain unmapped reads or reads that are mapped outside the targeted regions. In order to quantify the fraction of sequencing data that are not useful for variant calling within regions in the BED file, CoverView creates a chromosome-level summary that reports the total read counts (RC) and the read counts of on-target (RCIN) and off-target regions (RCOUT) for each chromosome. In addition, the outputted table includes the mapped, unmapped and total read counts calculated for the whole genome.

**Figure 1.**
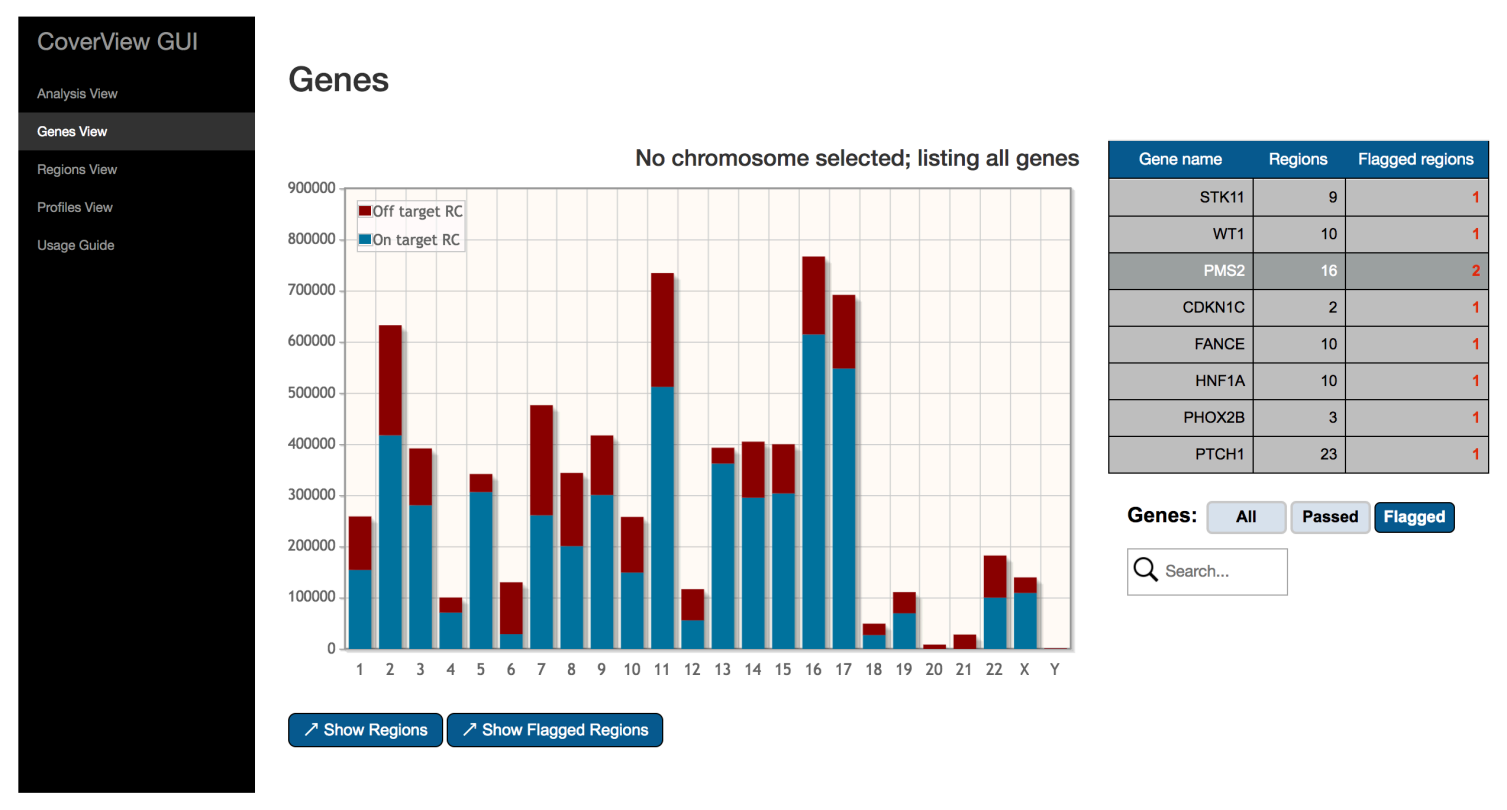
CoverView GUI Genes View for TSCP analysis in GIAB sample. CoverView GUI screenshot of the Genes View for TruSight Cancer Panel (TSCP) data generated for the Genome in a Bottle (GIAB) sample shows a bar plot of per-chromosome read counts and the list of flagged regions. Nine regions in eight genes were flagged for falling below MINQCOV ≥50 in the GIAB sample.

**Figure 2.**
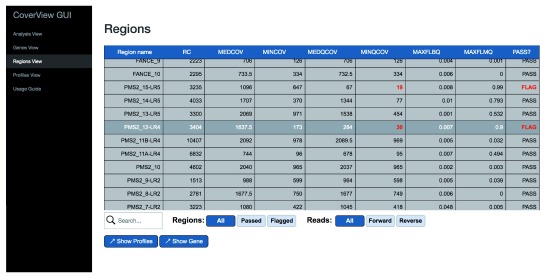
CoverView GUI Regions View for *PMS2* in GIAB sample. CoverView GUI screenshot of Regions View for *PMS2* data in the GIAB sample showing the summary metrics per region. Metrics that fall below user-defined thresholds are shown in red and flagged. The MINQCOV for *PMS2* exons 12 and 15 are below MINQCOV ≥50.

**Figure 3.**
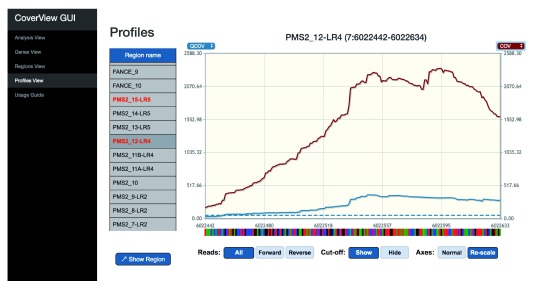
CoverView GUI Profiles View showing *PMS2* exon 12 COV and QCOV data in GIAB sample. CoverView GUI screenshot of Profiles View for *PMS2* exon 12 data in the GIAB sample showing the quality coverage QCOV (blue), per-base coverage COV (red) and the minimum QCOV threshold as dashed horizontal lines, across the region. The useful (quality) coverage is only a small proportion of the total coverage.

**Figure 4.**
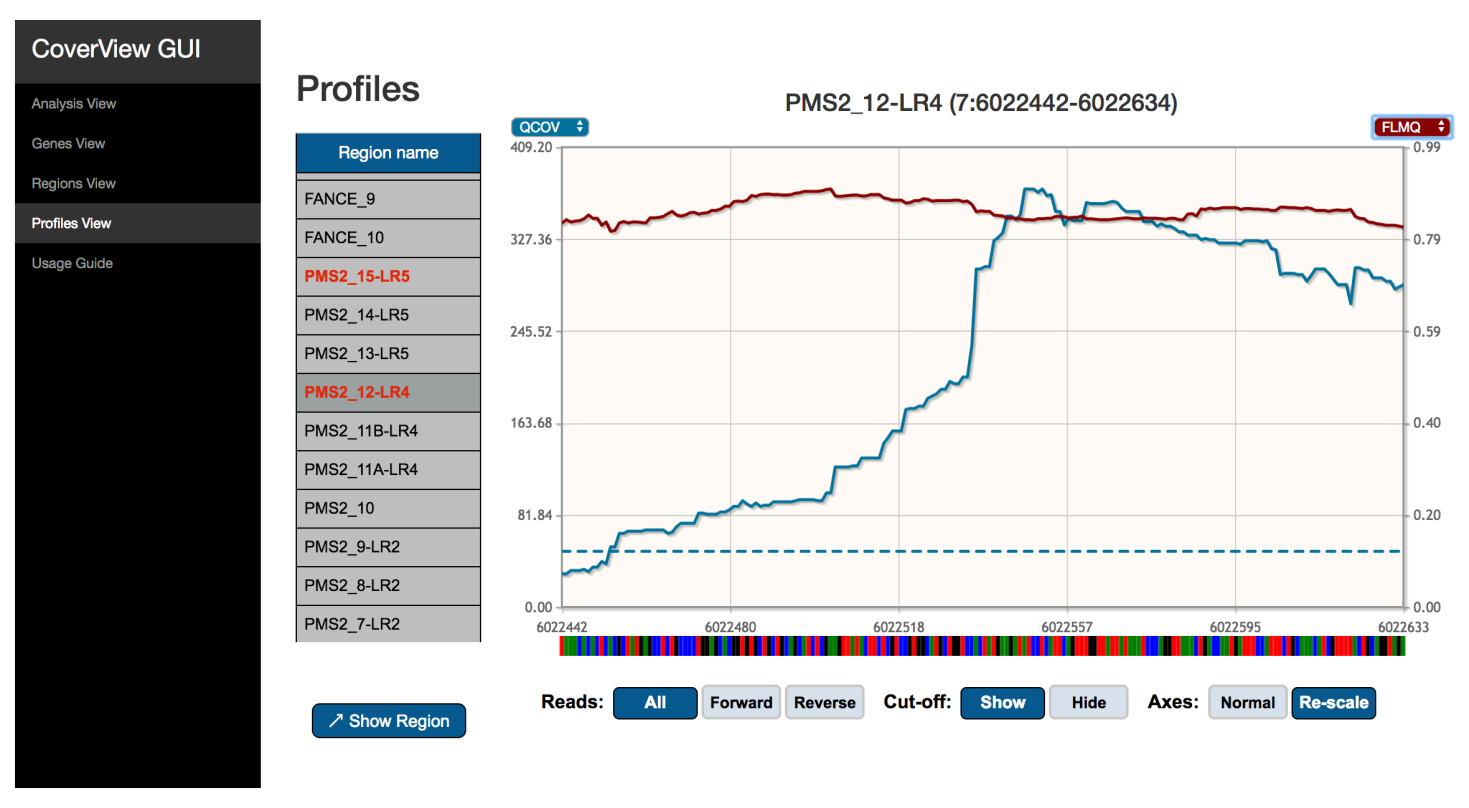
CoverView GUI Profiles View showing *PMS2* exon 12 QCOV and FLMQ data in GIAB sample. CoverView GUI screenshot of Profiles View for *PMS2* exon 12 data in the GIAB sample showing the quality coverage QCOV (blue), fraction of read bases with low mapping quality FMLQ (red) and the minimum QCOV threshold as dashed horizontal lines, across the region. This shows that the majority of reads mapping to this region have low mapping quality.

Finally, CoverView provides a GUI with multiple views (‘Analysis View’, ‘Genes View’, ‘Regions View’, ‘Profiles View’), that allow users to intuitively explore the results. The Analysis View shows metadata of the analysis such as the names of input files and key configuration options. The Genes View offers a clickable bar plot displaying per-chromosome read counts (both on-target and off-target) and a filterable table providing information on the number of flagged regions for each gene on the selected chromosome ( Figure 1). The Regions View displays a scrollable, searchable and filterable table of region-specific metrics values ( Table 2) with flagged regions and the metric(s) underlying the flag highlighted ( Figure 2). The Profiles View provides an interactive table and graph of per-base quality profiles for a selected region aligned with the corresponding reference genome sequence. Users can change the metrics displayed, zoom in, or navigate along the sequence with quality minimum thresholds overlaid as dashed horizontal lines ( Figure 3, Figure 4). The GUI is a Flask application that runs in the web browser on port 5000.

### Operation

CoverView can be installed by running a simple Bash script. Installation requires Python v.2.7.9 or later (Python2 series), GCC v.4.2.1, GNU make v.3.81 and virtualenv v.15.1.0 ^22^. Note that Python v.2.7.9 and later include *pip* by default. Additional dependencies (e.g. the Python module pysam ^23^) are automatically downloaded and set up in an isolated Python virtual environment by the installation script.

Once installed, the tool can be customised with a simple INI configuration file and run from Linux/Unix command line (see Documentation for details). CoverView can be easily integrated into NGS analysis pipelines, as shown for the OpEx (Optimised Exome) ^18^ pipeline. The CoverView documentation is hosted on GitHub Pages at https://rahmanteamdevelopment.github.io/CoverView/

CoverView v.1.4.3 documentation is also included in Supplementary File 1.

## Use case

We use the TruSight Cancer Panel (TSCP) for testing cancer predisposition genes in both research and clinical settings ^24– 26^. Here we generated TSCP data on the National Institute of Standards and Technology (NIST) Reference Material (RM) 8398, for which there are experimentally validated genotyped sites provided by the NIST-led Genome in a Bottle (GIAB) Consortium ^27^. We mapped paired-end Illumina sequencing reads to the human reference genome ( GRCh37) using Stampy v.1.0.20 ^28^ with BWA v.0.7.5a ^29^ for pre-mapping. Duplicate reads were marked with Picard v.1.90 ^30^. The resulting BAM file was analysed with CoverView v.1.4.3 with a BED file specifying the TSCP targeted regions. Duplicate reads were included in the analysis. In this use case we set the minimal requirement for a region to ‘PASS’ as MINQCOV ≥50: i.e. all base positions in a targeted region were required to be covered by at least 50 reads of good quality (MQ ≥ 20 and BQ ≥10). The CoverView analysis was completed in 13 seconds (for CoverView Output files see *Data and software availability* section) ^19^.

Nine of the 1471 targeted regions, affecting eight genes, were flagged as not fulfilling the MINQCOV ≥50 requirement ( Figure 1) ^19^. Here we focus on *PMS2* exon 12 as an example of how the CoverView GUI can help to investigate the underlying reasons of poor quality.

The Regions View provides region-level metrics values for *PMS2* exon 12 ( Figure 2). The table shows that 3404 reads were mapped to this region. Although the least covered base has 173 reads (MINCOV=173), one part of the region was covered with only 30 reads of good quality (MINQCOV=30). The MINQCOV column is therefore highlighted in red, indicating that its value is below the pre-set quality requirement. The table also suggests that the large difference between MINCOV and MINQCOV is due to poor mapping quality because the fraction of low mapping quality reads in the region is very high (MAXFLMQ=0.9; at a given position 90% of reads did not fulfill MQ ≥ 20). This is further supported by the Profiles View which shows the COV and QCOV metrics together ( Figure 3). These two depth of coverage profiles along the entire exon are substantially different, and the FLMQ profile shows that the mapping quality is consistently poor (FLMQ>0.8) along the whole exon ( Figure 4). This explains the striking difference between the COV and QCOV profiles since low quality reads are not counted as part of quality depth of coverage. *PMS2* has a nearby pseudogene with strong homology to exons 9, 11–15 that causes ambiguous mapping and it is not possible to robustly analyse exon 12 by TSCP data alone ^8^. However, the CoverView outputs show that every base in 1462/1471 (99%) TSCP regions in the GIAB sample pass the MINQCOV ≥50 quality threshold ^19^.

## Conclusion

Next generation sequencing data are error-prone, subject to random errors affecting individual samples and systematic errors, due to sequence contexts and biases of sequencing platforms, affecting many samples. Stringent, comprehensive quality management is therefore essential when using NGS for clinical applications. CoverView is a freely available NGS quality evaluation tool that provides quality metrics at the highest possible resolution by outputting per-base profiles, together with informative summary metrics that highlight which areas require further review. The CoverView outputs can be integrated into NGS pipelines so that regions that pass user-defined thresholds can automatically proceed and regions flagged as falling below user-defined thresholds can be further evaluated. The CoverView GUI provides a simple, visual interface with which to explore CoverView outputs and to investigate flagged regions. 

We developed CoverView to be easy to install and use and we believe it can be quickly integrated into any NGS pipelines. CoverView is now the quality evaluation tool we use for all our clinical and research NGS analyses.

## Data and software availability

CoverView input and output files for TSCP analysis in GIAB sample are available at: http://doi.org/10.17605/OSF.IO/87K6S ^19^

Data are available under the terms of the Creative Commons Zero "No rights reserved" data waiver (CC0 1.0 Public domain dedication).

CoverView is available at: github.com/RahmanTeamDevelopment/CoverView/releases and www.icr.ac.uk/CoverView

CoverView documentation is available at: https://rahmanteamdevelopment.github.io/CoverView/

Latest source code: https://github.com/RahmanTeamDevelopment/CoverView

Archived source code as at time of publication: http://doi.org/10.5281/zenodo.1206100 ^31^

Software license: MIT

## Supplementary material

**Supplementary File 1. CoverView v.1.4.3 documentation.**
