# Supplementary material for: CoverView: a sequence quality evaluation tool for next generation sequencing data
Source: Wellcome Open Res. 2018 Apr 4;3:36. [Version 1] doi: 10.12688/wellcomeopenres.14306.1 (PMC5964631; doi:10.12688/wellcomeopenres.14306.1)
Supplement: Supplementary file 1 [file wellcomeopenres-3-15567-s0000.tgz › 952de8f0-f896-4a61-9e9c-8e2c884f8510.pdf]

Table Of Contents

1. Introduction
2. Installation
   - 2.1. Dependencies
   - 2.2. Installation on Linux or Mac
   - 2.3. Installation on Windows
3. Running CoverView
   - 3.1. With BED file
   - 3.2. Without BED file
   - 3.3. The ensembl_db tool
4. Configuration file
5. Output files
   - 5.1. Chromosome level summary
   - 5.2. Per-base profiles
   - 5.3. Summary metrics of regions
   - 5.4. Poor quality intervals
6. Graphical User Interface (GUI)
   - 6.1. Analysis View
   - 6.2. Genes View
   - 6.3. Regions View
   - 6.4. Profiles View
7. CoverView paper

Previous topic

CoverView v1.4.3 documentation

This Page

Show Source

Quick search

Go

1. Introduction

CoverView is a fast, flexible and user-friendly coverage and base/mapping quality evaluation tool for panel and whole exome next-generation sequencing. It requires a BAM file containing the mapped reads and a BED file specifying the genomic regions for which coverage and the QC metrics will be reported. CoverView outputs QC metrics in multiple output files with increasing levels of detail from a chromosome level summary to per-base profiles. It also flags regions that do not pass pre-defined quality requirements. An interactive Graphical User Interface (GUI) running in the web browser is included for easier exploration of the QC results. For more details, read the CoverView paper.

2. Installation

2.1. Dependencies

To install and run CoverView v1.4.3 you will need the following dependencies installed:

- Python 2.7.9 or later (Python2 series)
- GCC and GNU make
- virtualenv

If your system is missing *GCC* and *GNU make*, these can be installed as follows: On a Mac, the easiest way to set them up is to install Xcode Command Line Tools. On a Debian or Ubuntu Linux, they can be set up by installing the *build-essential* package:

```
sudo apt-get install build-essential
```

If not already installed on your system, *virtualenv* can be set up by:

```
pip install virtualenv
```

2.2. Installation on Linux or Mac

CoverView v1.4.3 can be downloaded from GitHub here in either `.zip` or `.tar.gz` format. To unpack these run one of the following commands:

```
unzip CoverView-1.4.3.zip
```

or:

```
tar -xvzf CoverView-1.4.3.tar.gz
```

and then you can install CoverView with the following commands:

```
cd CoverView-1.4.3
./install.sh
```

CoverView uses *virtualenv* and *pip* to manage all its extra dependencies, which means that it will not clutter up your system by installing things globally. Everything it installs will go into a sub-directory in the `CoverView-1.4.3` directory. If you delete CoverView then everything it has installed will also be deleted. Once the installation script has finished successfully, CoverView is ready for use.

2.3. Installation on Windows

Installation on Windows is currently not supported.

3. Running CoverView

3.1. With BED file

Once correctly installed, CoverView can be run with the following simple command:

```
CoverView-1.4.3/coverview -c config.txt -i input.bam –b panel.bed -o example
```

or you can optionally supply a transcript database:

```
CoverView-1.4.3/coverview -c config.txt -i input.bam –b panel.bed -o example -t transcript_database.gz
```

By default, CoverView takes four command line arguments: the name of the configuration file (-c), the name of the input BAM file (-i), the name of a BED file (-b) and the output file name prefix (-o).

- The configuration file (-c) contains the user-specified settings (see the Configuration file section) and must follow the INI format
- The input BAM file (-i) must follow the BAM format containing the mapped reads with its .bai index file also present in the same directory. The BAM file may optionally contain reads marked as duplicates as CoverView can generate metrics with duplicate reads either included or excluded. The BAM file must contain reads/read groups from only a single sample.
- The BED file (-b) must follow the BED format with each record corresponding to a region of interest (e.g. exon)
- The transcript database (-t) is optional; it must be generated by the `ensembl_db` tool (see The ensembl_db tool section)

3.2. Without BED file

The -b command line flag is optional. If a bed file is not specified, only a simplified chromosome level read count summary will be outputted:

```
CoverView-1.4.3/coverview -c config.txt -i input.bam -o example
```

3.3. The ensembl_db tool

If transcript coordinates are to be outputted, CoverView requires a transcript database file. The `ensembl_db` tool can be used to generate the transcript database file for an arbitrary Ensembl release.

For example, you can generate a transcript database file based on Ensembl release 75 by running

```
CoverView-1.4.3/ensembl_db -e 75 -o output
```

where -e specifies the Ensembl release version and -o sets the output file name prefix. The resulting *output.gz* file can be supplied to CoverView by its -t command line option (see Running CoverView).

Alternatively, an input text file containing a list of Ensembl ENST identifiers (a single ID per each line) can be specified in order to include only the listed transcripts in the database:

```
CoverView-1.4.3/ensembl_db -i input.txt -e 75 -o output
```

4. Configuration file

The CoverView configuration file uses the INI format. As illustrated by the example below, the configuration file may contain five sections: [reads], [outputs], [transcript], [quality] and [pass].

```
[reads]
duplicates = true
direction = false

[outputs]
regions_file = true
profiles_file = true
only_flagged_profiles = false

[transcript]
regions_file = false
profiles_file = false

[quality]
low_bq = 10
low_mq = 20

[pass]
MINQCOV_MIN = 15
```

The following options may be specified in the configuration file

Section	Key	Type	Default	Effect
reads	duplicates	Boolean	true	if true then duplicate reads are included in the analysis
reads	direction	Boolean	false	if true then per-region metrics and per-base profiles are also output for forward and reverse-stranded reads separately
outputs	regions_file	Boolean	true	if true then the _regions.txt output file will be written
outputs	profiles_file	Boolean	true	if true then the _profiles.txt output file will be written
outputs	only_flagged_profiles	Boolean	false	if true then the _profiles.txt output file will contain flagged regions only
transcript	regions_file	Boolean	false	if true then transcript coordinates are reported in the _regions.txt file
transcript	profiles_file	Boolean	false	if true then transcript coordinates are reported in the _profiles.txt file
quality	low_bq	Integer	10	base quality cut-off used in the FLBQ metrics
quality	low_mq	Integer	20	mapping quality cut-off used in the FLMQ metrics
pass	?_MIN / ?_MAX	Integer	none	requirements a region must satisfy to be labelled as *PASS*

The [pass] section specifies a set of one or more requirements a region must satisfy in order to be labelled as *PASS* in the output, otherwise the region will be *flagged*. Each requirement is given as a key-value pair where the key should follow the format of METRIC_MIN (to set a minimum requirement) or METRIC_MAX (to set a maximum requirement). METRIC can be any of the per-region metrics defined in 5.3 (Summary metrics of regions). For example, the following specifies that regions with MINQCOV<15 are to be flagged:

```
[pass]
MINQCOV_MIN = 15
```

This second example specifies that regions with MINCOV<30 or MAXFLBQ>0.2 are to be flagged:

```
[pass]
MINCOV_MIN = 30
MAXFLBQ_MAX = 0.2
```

5. Output files

CoverView generates 4 easily parsable (column-based) output files:

- <prefix>_summary.txt (chromosome level summary)
- <prefix>_profiles.txt (per-base profiles)
- <prefix>_regions.txt (summary metrics of regions)
- <prefix>_poor.txt (poor quality intervals)

where <prefix> denotes the output file name prefix specified by the -o command line option. (Note that an additional file, <prefix>_meta.txt, is also created that is required by the GUI.)

5.1. Chromosome level summary

The *_summary.txt* output file provides chromosome level summary (read counts) and contains the following 4 columns:

**Column name**	**Description**
CHROM	chromosome name
RC	total read count; total number of reads mapped to the chromosome
RCIN	read count in targeted regions; number of reads mapping to the chromosome that overlap targeted regions from the BED file
RCOUT	read count outside of targeted regions; number of reads mapping to the chromosome that do not overlap targeted regions from the BED file

In addition to the list of chromosomes, the outputted table also reports the mapped, unmapped and total read counts for the whole dataset.

5.2. Per-base profiles

The *_profiles.txt* output file provides per-base profiles for the targeted regions. Each position is described in a separate line with the following 8 columns:

**Column name**	**Description**
Chromosome	chromosome name
Position	position on chromosome
COV	coverage; number of reads covering the position
QCOV	quality coverage; number of reads covering the position with a MQ larger than the threshold set by the `low_mq` configuration flag and a BQ larger than the cut-off set by the `low_bq` flag
MEDBQ	median base quality; median base quality of all read bases mapping to the position
FLBQ	fraction of low base quality; fraction of read bases mapping to the position with a BQ smaller or equal than the cut-off set by the `low_bq` configuration flag
MEDMQ	median mapping quality; median mapping quality of all reads covering the position
FLMQ	fraction of low mapping quality; fraction of reads covering the position with a MQ smaller or equal than the cut-off set by the `low_mq` configuration flag

An additional column named “Transcript_coordinate” is included in the output if the `profiles_file` flag is set to *true* in the [transcript] section of the configuration file. This column provides the transcript coordinate of the position with regards to the overlapping transcript. In case the position overlaps with multiple transcripts, the coordinates in all transcripts are reported separated by commas. Transcripts data are read from the user-specified transcript database (see Running CoverView).

Finally, if the `direction` flag is set to *true* in the [reads] section of the configuration file, 12 additional columns are added to the *_profiles.txt* file:

- `COV+`, `QCOV+`, `MEDBQ+`, `FLBQ+`, `MEDMQ+` and `FLMQ+`: the same metrics as `COV`, `QCOV`, `MEDBQ`, `FLBQ`, `MEDMQ` and `FLMQ` defined above, however, considering only forward-stranded reads
- `COV-`, `QCOV-`, `MEDBQ-`, `FLBQ-`, `MEDMQ-` and `FLMQ-`: the same information, considering only reverse-stranded reads

5.3. Summary metrics of regions

The *_regions.txt* output file provides a number of metrics summarizing the per-base profiles of each region. These summary metrics give information on the overall quality of each region. In addition, regions are marked as *"PASS"* or *"FLAG"* based on the requirements set in the configuration file. Each line in the file corresponds to a region described by the following 12 columns:

Column name	Description
Region	region name taken from the 4th column of the BED file
Chromosome	chromosome name
Start_position	start position of region on chromosome
End_position	end position of region on chromosome
Pass_or_flag	'PASS' if region satisfies the requirements set in the [pass] section of configuration file or 'FLAG' otherwise
RC	read count; total number of reads overlapping with the region
MEDCOV	median coverage; median COV values across all positions in the region
MINCOV	minimum coverage; minimum of COV values across all positions in the region
MEDQCOV	median quality coverage; median of QCOV values across all positions in the region
MINQCOV	minimum quality coverage; minimum of QCOV values across all positions in the region
MAXFLMQ	maximum fraction of low mapping quality; maximum of FLMQ values across all positions in the region
MAXFLBQ	maximum fraction of low base quality; maximum of FLMB values across all positions in the region

Note: If there are multiple regions in the BED file with the same name in their 4th column (e.g. the regions correspond to different exons of the same gene), CoverView adds an index to the region names joined by an underscore. For example, multiple regions of the BRCA2 gene would be referred to as BRCA2_1, BRCA2_2, BRCA2_3 etc.

Two additional columns named "Start_transcript" and "End_transcript" are included in the output if the `regions_file` flag is set to *true* in the [transcript] section of the configuration file. These columns provide the transcript coordinates of the start and end positions of the region with regards to overlapping transcripts.

Finally, if the `direction` flag is set to *true* in the [reads] section of the configuration file, 12 additional columns are added to the *_regions.txt* file:

- `MEDCOV+`, `MINCOV+`, `MEDQCOV+`, `MINQCOV+`, `MAXFLMQ+` and `MAXFLBQ+`: the same metrics as `MEDCOV`, `MINCOV`, `MEDQCOV`, `MINQCOV`, `MAXFLMQ` and `MAXFLBQ` defined above, however, considering only forward-stranded reads
- `MEDCOV-`, `MINCOV-`, `MEDQCOV-`, `MINQCOV-`, `MAXFLMQ-` and `MAXFLBQ-`: the same information, considering only reverse reads

5.4. Poor quality intervals

If the `profiles_file` option is set to *true* in both the [outputs] section and [transcript] section of the configuration file, CoverView will create a fourth output file; *<prefix>_poor.txt*. This file provides a comprehensive list of all continuous intervals within the studied regions with QCOV<15 for all bases (referred to as *"poor quality intervals"*). Note that multiple such intervals may exist in a single region. Each line in the file corresponds to a *poor quality interval* with the following 6 columns:

Column name	Description
Region	name of region which contains the interval
Chromosome	chromosome name
Start_position	start position of interval on chromosome
End_position	end position of interval on chromosome
Start_transcript	start coordinate of interval in the overlapping transcript
End_transcript	end coordinate of interval in the overlapping transcript

In case the start or end position overlaps with multiple transcripts, the coordinates in all transcripts are reported separated by commas.

6. Graphical User Interface (GUI)

CoverView provides an interactive GUI that allows visual exploration of the QC results. The GUI can be run from the terminal importing a CoverView output prevously generated for a sample:

```
CoverView-1.4.3/gui -i /path/to/data/example -r /path/to/reference/reference.fasta
```

The GUI opens in the web browser (*Chrome*, *Safari* and *Firefox* are supported).

Mandatory command line options are:

- -i specifies the input file name prefix (i.e. *example_regions.txt*, *example_profiles.txt*, *example_summary.txt* etc. files should be in the directory /path/to/data/)
- -r specifies the reference genome FASTA file. Note that the reference genome file must be indexed by samtools faidx and the .fai index file has to be in the same directory as the FASTA file. The genome build must be the same as used for creating the BED file

Note that in order to be accepted by the GUI, the CoverView output are required to be generated with the following configuration settings:

```
[outputs]
regions_file = true
profiles_file = true
only_flagged_profiles = false
```

The CoverView GUI consists of the following four views discussed in the next subsections:

- Analysis View
- Genes View
- Regions View
- Profiles View

6.1. Analysis View

The *Analysis View* provides a summary table of sample name, input files, key configuration options and the date of running the CoverView analysis. The displayed sample name is extracted from the read group (@RG) lines of the header of the BAM file, if the information is present. In addition, the total number of *flagged* regions and genes are also presented. (A gene is *flagged* if it includes at least one *flagged* region.) If a text is too long to be displayed in the table, hover the mouse cursor over it to see the full text in the tooltip.

6.2. Genes View

The *Genes View* provides a bar plot displaying per-chromosome read counts and a table at the right side showing the total number of regions and the number of *flagged* regions for each gene. The bar plot presents both on-target and off-target read counts for each chromosome. Clicking on a particular bar in the plot selects/unselects the chromosome. If a chromosome is selected, only the genes located on that chromosome are listed in the table, otherwise all genes are shown.

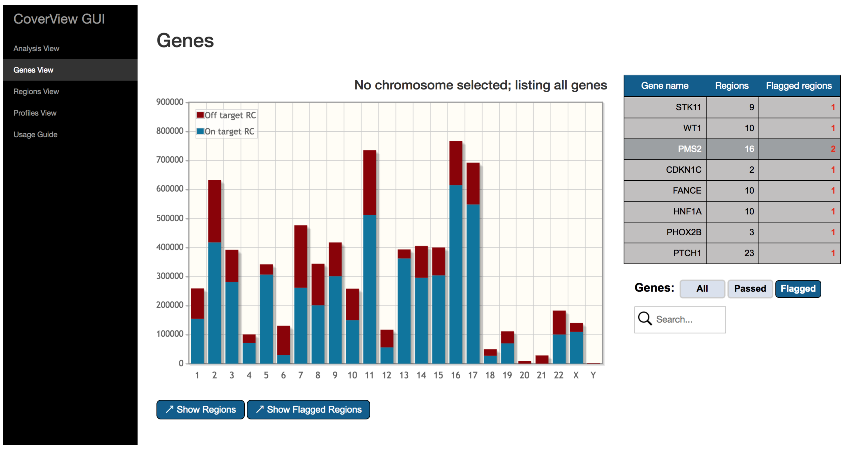

Flagged genes (i.e. those with at least one *flagged* region) are highlighted in red. The table can be filtered to show either passed, flagged or all genes by the “Genes” option. The search box helps to find particular genes of interest.

A gene can be selected/unselected by clicking on the row of the table. If a gene is selected, the “Show Regions” and “Show Flagged Regions” buttons are visible. With “Show Regions” you can open the selected gene in *Regions View* where only the regions of the gene will be displayed. Clicking on the “Show Flagged Regions” button also redirects to *Regions View*, but only *flagged* regions of the gene will be listed.

6.3. Regions View

The *Regions View* provides a scrollable table of the genomic regions that have been analysed by CoverView. Columns of the table provide the following per-region metrics: `RC`, `MEDCOV`, `MINCOV`, `MEDQCOV`, `MINQCOV`, `MAXFLBQ`, `MAXFLMQ` and `PASS?`. For *flagged* regions, metrics values not satisfying quality requirements are highlighted in red.

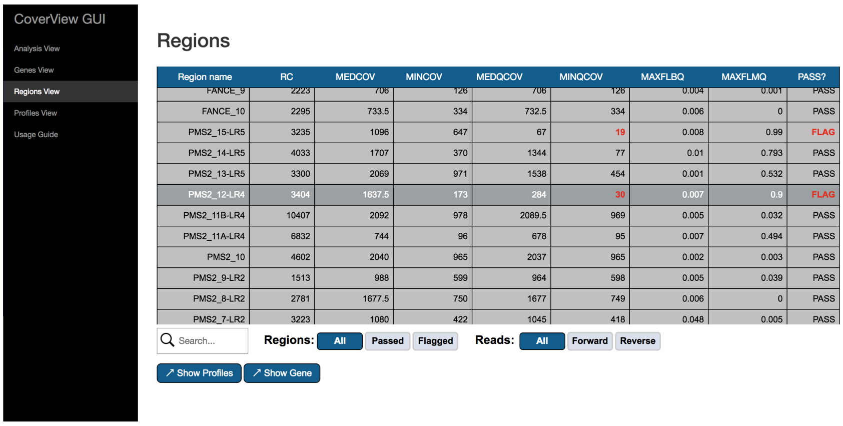

The table can be filtered to show either passed, flagged or all regions by using the “Regions” option. With the “Reads” option, you can switch between displaying the above metrics for forward reads only, reverse reads only or all reads included. The search box helps to find particular regions of interest.

A region can be selected/unselected by clicking on the row of the table. If a region is selected, the “Show Profiles” and “Show Gene” buttons are visible. With “Show Profiles” you can open the selected region in *Profiles View*. Clicking on the “Show Gene” button redirects to *Gene View* showing the gene to which the selected region belongs.

6.4. Profiles View

The *Profiles View* provides an interactive graph of per-base quality profiles aligned with the reference sequence. The visible profiles correspond to the region selected in the left-side panel listing all regions with *flagged* regions highlighted in red. The diplayed metrics can be changed with the selector at the top of both y-axes. The following per-base metrics can be selected: `COV`, `QCOV`, `FLBQ`, `MEDBQ`, `FLMQ` and `MEDMQ`. If two metrics are plotted together, the color of each line is the same as of the corresponding metrics selector (blue for the y-axis and red for the y2-axis). Hover the mouse cursor over a point of the plot to see the exact genomic position and metric value.

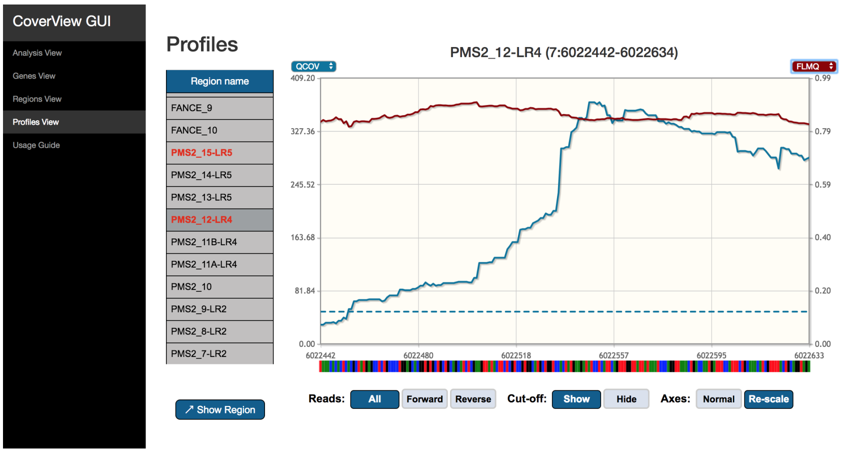

Click and drag on the plot to zoom in any section of the profiles. The genomic coordinates of the zoomed region are provided under the graph. You can move upstream and downstream or zoom out with the navigation buttons. It is possible to repeatedly zoom in up to DNA base-level. If the “Axes” option is set to “Normal”, the y-axes will have the same scale when you zoom in, otherwise they will be re-scaled to be optimised for the visible section of the profiles.

With the “Reads” option, you can switch between showing the per-based profiles for forward reads only, reverse reads only or all reads included. Quality cut-off values specified by the [pass] section of the CoverView configuration file can be displayed as dashed horizontal lines by switching on the “Cut-off” option. A horizontal line representing the cut-off value is visible only if the relevant metric is displayed and have the same color.

Clicking on the “Show Region” button opens the selected region in the *Region View*.

7. CoverView paper

Link to CoverView paper coming soon.

© Copyright 2018, Rahman Lab. Created using Sphinx 1.6.3.
