# Supplementary material for: CoverView: a sequence quality evaluation tool for next generation sequencing data
Source: Wellcome Open Res. 2018 Apr 4;3:36. [Version 1] doi: 10.12688/wellcomeopenres.14306.1 (PMC5964631; doi:10.12688/wellcomeopenres.14306.1)
Supplement: Peer review history

# Peer review: Referee response for version 1

**DOI:** 10.21956/wellcomeopenres.15567.r32998

**Published:** 2018-05-22

**Authors:** Somak Roy

**Affiliations:** 1 Department of Pathology, Division of Molecular & Genomic Pathology, University of Pittsburgh, Pittsburgh, PA, USA

The authors have described their software solution, CoverView, to analytically and visually profile Next Generation Sequencing (NGS) data for use in a clinical environment. Specifically, the manuscript describes quality control parameters derived from sequencing depth, base quality, and mapping quality score.

 The described software solution appears to have great utility as a pluggable tool in the clinical workflow of QC review of NGS data. The graphical user interface (GUI) appears to be intuitive and user friendly for use by laboratory personnel who may not have experience with command line and other programming tools. This tool can potentially be a useful resource for validation of NGS assays where understanding the coverage and sequencing quality profile across sequenced region is important.

 I summarize my comments below that should help further improve the content of the manuscript.

1. The authors have described sequencing depth, base quality, and mapping quality scores as the primary measurements for CoverView. However, the drafted manuscript reads such that these three parameters are the only indices to be evaluated for quality. Since there are additional parameters from sequenced reads that reflect quality of NGS data ^1^, the authors should list these under discussion section of the manuscript for the readers to have a holistic view of NGS QC.
2. Scalability: the manuscript demonstrates the application performance (computational time, visual rendering, and user interaction) based on using data from targeted panel. Parsing and analyzing data from BAM files can be computationally expensive, especially as the sequenced region increases (targeted panels with several hundred genes, exomes, etc). The authors reference to integrating CoverView in their clinical exome sequencing pipeline (OpEx). May I request the authors to provide benchmark for computational time, visual rendering, and user interaction comparing OpEx and TSCP to demonstrate the scalability of the software solution. It will be interesting to know how the rendered user interface adapts to large array of data points while preserving user experience.
3. Input BAM file and multiple aligners: Can the authors discuss their experience with regards to the input BAM file when generated by multiple sequence alignment algorithms? In the use case, the authors describe using Stampy and BWA. I am assuming that each of the BAM files were separately analyzed by CoverView. Did the authors face any challenges when using BAM files from different alignment algorithms?
4. Quality Sequencing Minimum (QSM): The authors reference QSM as being used with CoverView in their laboratory. It will be helpful if some description of QSM is included in the manuscript.

I have read this submission. I believe that I have an appropriate level of expertise to confirm that it is of an acceptable scientific standard.

## References

[1] RoySColdrenCKarunamurthyAKipNSKleeEWLincolnSELeonAPullambhatlaMTemple-SmolkinRLVoelkerdingKVWangCCarterAB: Standards and Guidelines for Validating Next-Generation Sequencing Bioinformatics Pipelines: A Joint Recommendation of the Association for Molecular Pathology and the College of American Pathologists. .2018;20(1) : 10.1016/j.jmoldx.2017.11.003 4-27 10.1016/j.jmoldx.2017.11.003 29154853

# Peer review: Referee response for version 1

**DOI:** 10.21956/wellcomeopenres.15567.r32740

**Published:** 2018-04-20

**Authors:** Richard Bagnall

**Affiliations:** 1 Agnes Ginges Centre for Molecular Cardiology, Centenary Institute, Sydney Medical School, The University of Sydney, Sydney, NSW, Australia

Coverview is presented as a computational tool to assess the amount, and quality, of aligned next generation sequencing data reads at user-defined regions. Additionally, a data visualisation tool that runs in a web-browser window displays the results and allows the user to navigate the data and investigate regions that are flagged as failing a quality metric. Coverview is designed with clinical laboratories in mind, where the assurance that target regions have been adequately sequenced is vital. The tool and underlying computer code is made freely available, and a detailed user manual is provided.

 A number of tools and approaches are already available to measure and visualise the amount and quality of NGS read data, as these are important factors in determining the reliability, sensitivity and specificity of variant calling. However, Coverview brings the analysis and visualisation of results together in a single package, requires very basic knowledge of the command line, and should be a useful addition.

 The manuscript is clear and presents the features of Coverview, with presentation of a use case using a validated gene panel (TruSight Cancer Panel). This use case shows how high depth sequencing of an established gene panel yields only a few regions of low coverage for interrogation. Although, in principal, Coverview could be used to investigate an exome, in practice the number of flagged regions may become unwieldy.

 General comments:

 A description of the QSM would be helpful, as most people will not know what this is.

 The number of bases below QCOV for each region on the ‘Regions View’ is not displayed, but could help the user prioritise regions for further interrogation.

 Thank you for introducing CoverView.

I have read this submission. I believe that I have an appropriate level of expertise to confirm that it is of an acceptable scientific standard.
